# 2636. Discovery of Spike Identification (SpiID) Nanobody Panel for SARS-CoV-2 Vaccine Development

**DOI:** 10.1093/ofid/ofad500.2248

**Published:** 2023-11-27

**Authors:** Qi Li, Jennifer Doering, Zepei Ma, Nicholas Mantis, Lisa Cavacini, Yang Wang

**Affiliations:** MassBiologics, Boston, Massachusetts; Wadsworth Center, Albany, New York; MassBiologics, Boston, Massachusetts; Wadsworth Center, Albany, New York; MassBiologics, Boston, Massachusetts; MassBiologics, Boston, Massachusetts

## Abstract

**Background:**

SARS-CoV-2 variants of concern (VoC), particularly the Omicron lineage, are spreading rapidly throughout the world even among vaccinated populations. Unfortunately, the first-generation vaccines and monoclonal antibody therapeutics have significantly reduced efficacy against new variants, creating a need for multivalent next-generation countermeasures, including spike protein-based subunit vaccines.

**Methods:**

Although spike protein-based subunit vaccines are relatively easy and cost-effective to manufacture, the challenges present when it comes to Chemistry, Manufacturing, and Controls (CMC) from a clinical perspective. Specifically, multivalent and/or combination subunit vaccines of different variants require specific identity testing with analytical assay reagents which can distinguish closely related antigens. In order to overcome this difficulty, a panel of SARS-CoV-2 VoC-specific nanobodies was developed from a diverse synthetic yeast-display library.

**Results:**

These nanobodies, when expressed with human IgG-Fc fragments as fusion antibodies, showed high specificity against individual variants with nanomolar range affinity (8.1- 68nM) against various VoCs, including Wuhan, Beta, Delta, and Omicron with no cross-reactivity with other variants. Additionally, a subset of nanobodies demonstrated potent virus-neutralizing activity *in vitro* in the sub-nanomolar range (0.1-0.8nM), and may be useful as biological standards in clinical settings.

**Conclusion:**

These findings offer potential CMC solutions for developing multivalent and/or combination subunit vaccines against newly emerged SARS-CoV-2 variants, improving clinical outcomes for patients.

**Disclosures:**

**All Authors**: No reported disclosures

